# Intervening on impostor phenomenon: prospective evaluation of a workshop for health science students using a mixed-method design

**DOI:** 10.1186/s12909-022-03824-7

**Published:** 2022-11-17

**Authors:** Shine Chang, Hwa Young Lee, Cheryl Anderson, Kava Lewis, Devasmita Chakraverty, Melinda Yates

**Affiliations:** 1 Department of Epidemiology, Unit 1365, Division of Cancer Prevention and Population Sciences, The University of Texas MD Anderson Cancer Center, 1155 Pressler Street, Houston, TX 77230-4009 USA; 2 Cancer Prevention Research Training Program, Division of Cancer Prevention and Population Sciences, The University of Texas MD Anderson Cancer Center, Houston, TX USA; 3 Ravi J. Matthai Centre for Educational Innovation, Indian Institute of Management Ahmedabad, Ahmedabad, Gujarat India; 4 Department of Gynecologic Oncology and Reproductive Medicine, Division of Surgery, The University of Texas MD Anderson Cancer Center, Houston, TX USA

**Keywords:** Impostor phenomenon, Growth mindset, Summer program, Intervention, Mixed-method

## Abstract

**Background:**

Unaddressed impostor feelings that impede developing interest in science and self-efficacy in conducting research have a dispiriting effect that perpetuates unsatisfactory diversity in the health science workforce when such feelings are experienced more by those historically underrepresented in the workforce. This warrants effective interventions to reduce the impact of impostor feelings and related factors that diminish career resilience. We examined the effects of a 90-minute workshop on impostor perceptions and growth mindset to raise awareness of impostor phenomenon (IP) and develop skills to manage IP successfully for students attending a 10-week summer research experience program.

**Methods:**

Using a convergent mixed-methods design, data were analyzed from 51 racially and ethnically diverse students who participated in an interactive IP workshop. Using students’ half-way and final progress reports about their summer experiences and pre- and post-summer online surveys, we identified how the workshop changed awareness of IP and helped students develop coping strategies.

**Results:**

Students strongly endorsed the workshop, remarking that its content and personal stories from peers validated their own IP experiences and relieved anxiety by revealing how common the experience was. Many reported applying mindset-changing solutions, including positive self-talk, focusing their thinking on facts about themselves and situation, and grounding themselves firmly against potentially persuasive and confidence-eroding impostor feelings. While students reported end-of-summer impostor feelings at levels similar to before the program, they described being able to manage their feelings better and persist towards goals and challenging tasks. One measure of IP appeared to be addressed through students’ activation of a growth mindset, potentially explaining a specific mechanism for intervention. Discrepancies between qualitative responses and quantitative IP measures demand additional work on IP instruments.

**Conclusions:**

A brief, theory-based IP workshop administered by research training programs, including those as short as 10-weeks, can have positive impact on subsequent IP experience and its successful management, with potential long-term impact on retention of a diverse biomedical research workforce.

For decades, significant resources invested by the National Institutes of Health (NIH) and others have aimed to foster a workforce of researchers diverse at all levels of training and career by gender, race, ethnicity, disadvantaged background, disability, and other factors [1], with limited success. Many factors influence entry into training and education in STEM fields, as well as exit from it when career resilience and interest flag. Departing trainees from groups historically underrepresented in biomedical research further diminish diversity in research, and more emphasis on institutional culture change is needed [2]. Research education and training programs funded by the NIH play a major role in addressing this problem [3, 4]. These programs provide students considering medical school or graduate research programs with opportunities to explore science, conduct research with mentors serving as role models engaged in fulfilling work, have experiences that position them to compete well for future training, and confirm the fit of chosen career paths.

For individuals from groups underrepresented in science and research (URM), such skill development may have key relevance for career persistence. Some have argued that clinical and research training environments, as well as academic health center environments, foster self-doubt [5] and fail to promote a sense of belonging, especially for women and URM groups [6]. Qualitative reports reveal feelings of being an impostor are common and recurrent, even among senior faculty physicians [5]. Impostor phenomenon (IP), characterized by feelings of intellectual or professional fraudulence and intense fear of being exposed as incompetent in spite of verified achievement, was first conceptualized in the 1970s [7]. Typically experienced when competent people doubt their competence, and do not view their success as a result of their ability and effort, Impostors fear being perceived as frauds who have fooled others into believing that they have high levels of ability. Over time, research on IP has focused on research doctoral students and postdoctoral fellows [8, 9], in medical [10] and dental students [11], and during preclinical to clinical transitions among medical students [12]. IP has also been studied in relation to burnout among medical students [13] and surgeons/residents [14]; to lack of well-being among pharmacy residents [15]; to increased distress and lower self-compassion and self-esteem in medical students [16] and among early-career palliative care clinicians [17].

Academic medicine has been suggested to foster self-doubt through conceptions of ability mindset [5], of which research has identified two major types: a *growth mindset*, where ability, skill, and performance on a task are viewed as acquirable through effort and practice; and a *fixed mindset*, where ability is viewed as a genetically derived, inherent and unalterable capacity [18–20]. Decades of research have found that those with growth (vs. fixed) mindsets adopt learning goals, seek challenging tasks, and regard failure as natural and instructive parts of the process of acquiring knowledge [21]. Recent work on IP and ability mindset [22] highlights the need to understand and intervene on factors related to trainees, their training environments, and factors that trigger impostor feelings, as these threaten persistence in clinical and research careers. Trainees considering careers in medicine and research, and those already committed to them both need to be able to strengthen their identities as scientists and clinicians, activate growth mindsets [23], manage perceived discrimination, including microaggressions [24], and cope with impostor feelings, as these may influence trainees’ sense of belonging in science and career resilience. Thus, recognizing the critical role of such skills in clinical and research careers and the need to master them is central both for recruiting trainees to the biomedical science workforce and reducing their departure from it. Delivering structured educational sessions to learn and practice managing and coping with such issues may be substantially better than relying on trainees to haphazardly acquire such skills themselves. To date, however, few evaluated interventions for managing IP are available [22, 25].

Given documented career benefit from participating in research during early career training [26–28], developing interventions to address IP early in training is urgent. We describe a brief interactive workshop to address impostor feelings and ability mindset delivered early in a 10-week, full-time, NCI/NIH-funded summer research experience program for college, graduate, and health professional students. We evaluated whether students improved their management of impostor feelings and used strategies to invoke growth mindsets, using both qualitative and quantitative evaluations. Specific questions were:

- Were there pre-post workshop changes in mean ratings for impostor feelings and growth mindset, and did changes differ by demographic groups?
- Were impostor ratings related to mindset ratings, and did the relationships change over time following the workshop?

We conclude with recommendations for developing interventions, delivered at critical junctures for career decisions and identity formation, to improve persistence in research careers in academic medicine.

## Method

### Participants

The University of Texas MD Anderson Cancer Center’s Cancer Prevention Research Training Program (CPRTP, www.CancerPreventionTraining.org/Summer) annually selects 25 nationally recruited undergraduate, graduate, and health profession students for mentored research experiences. The 10-week, multi-disciplinary program exposes students to topics and research skills used in cancer prevention, including bench-based methods, qualitative and community-based methods, statistical and other quantitative methods, as well as professional development skills, and career exploration [29]. Activities culminate in a poster exposition and student elevator speeches at the end of the summer.

We analyzed data from 51 students in two summer cohorts (2018, 2019) that included 35 women (69%), 16 men (31%), 16 Asians (31%), 12 African Americans (24%), 6 Latinx (12%), and 16 Whites (31%). In addition to 24 (47%) college students were 7 (14%) medical students and 20 (39%) masters and doctoral students. Demographic data were collected in the online application to the summer program. For gender, the fields were labeled, “gender” with the response categories: female, male, other.

### Ethics approval and consent to participate

This evaluation was approved as exempt and a waiver for informed consent was also approved by the Institutional Review Board (IRB) of The University of Texas MD Anderson Cancer Center (IRB #2020–0088 exemption).

### IP workshop

Expanded from a well-rated 30-minute presentation about IP, the 90-minute workshop was designed to help students by anticipating common challenges when entering the professional research environment at MD Anderson. Delivered during orientation each summer, the workshop focuses on IP and the importance of expecting and embracing uncertainty as a necessary component of impactful research. Small-group activities, facilitated large-group discussions, and individual work fostered interaction between students and deepened development of IP coping skills.

The workshop (Table 1) begins with a 3-minute essay (“what does it mean for you to feel like an ‘impostor’?”) and group discussion of the importance of “stupidity” and “failure” in research [30, 31]. These activities increase awareness about the role of these constructs in the scientific process with recognition of key but unspoken expectations or beliefs encountered or held by students during their transition from classrooms to research labs. This is followed by an introduction to IP and small group activities for students to practice identifying sources and triggers for IP, and then brainstorming strategies for coping with IP, which increases resilience by facilitating more rapid response to future IP challenges when they arise. After small group activities, groups share salient points in facilitated discussion with all students. The workshop concludes with discussion to define what “success” looks like (e.g., not a lack of failure) and the many qualities of successful scientists, using examples of Nobel laureates describing impostor experiences, and encouraging students to explore these issues with scientists encountered over the summer.

**Table 1:** Research Success & Survival Workshop for Trainees during CPRTP Orientation

Workshop Section	How the section is designed to achieve learning objectives
What does it mean for you to feel like an “impostor”? (**3-minute essay**)	Prime students’ mindset about IP; explain that researchers are trained to be pioneers and commonly feel different emotions, but the goal is to persist!
Doing research requires “stupidity”^30^ and “failure”^31^	Acknowledge situations as possible opportunities for IP (i.e., acceptance into prestigious program with other highly achieving students); excellence in research requires embracing uncertainty; normalize feelings of “stupidity” and experience of failure and rejection as routine in science and research exploration, even beneficial and necessary to do impactful research.
Benefits of failure/rejection (**individual brainstorm & share responses**)	Explore new perspectives of failure, rejection; guide them to derive benefit from failure, how to get the most from feedback; approach as process of learning.
Impact of impostor phenomenon (“why do we care about IP?”)	Define IP; **group brainstorm activity** to discuss why it is important to know about IP (connect with barriers to learning, goal achievement, career advancement). Students explain in their own words how IP might have impact on them or their peers.
Impostor phenomenon sources and triggers (**small group activity**)	To identify sources and triggers for IP, trainees share experiences and identify themes (evaluation/competition, prestigious programs, “high powered” researchers, experiencing success/seeing other trainees’ success).
Anti-impostor phenomenon strategies (**small group activity**)	Identify alternative approaches to sources/triggers they identified earlier to prepare for future IP experiences. [Acknowledge having areas for improvement does not mean being a fraud, being wrong or saying “I don’t know” isn’t catastrophic, know how common IP experiences really are, etc.]
1) Re-define success for yourself; and 2) Qualities of successful scientists	Students are challenged to recognize that being successful is not about lack of failure (citing “failures” from Nobel laureates). Ask trainees to define what they mean by research success (impact, advancing science, etc.). Encourage use of informational interviews to explore how senior researchers define success for themselves.

### Measures and procedure

Data were collected from online surveys self-administered 2 weeks before and at the end of each summer, and half-way and final progress reports. Surveys included one adapted IP item from the Impostor Phenomenon Scale by Harvey and Katz [32] and two items from Leary’s IP scale [33] and growth mindset items adapted from seminal work on mindset [20, 34]. The IP items were 1) “People tend to believe I am more competent than I really am,” (Harvey and Katz Item 1, dropping “in general” to allow reference to pre- and post-summer time points), 2) “I tend to feel like a phony,” (Leary Item 2), and 3) “Sometimes I’m afraid others will discover how much knowledge or ability I really lack,” (Leary Item 5). Ratings used Likert scales, ranging from 1 (not at all true of me) to 5 (completely true of me). The mindset items were 1) “Becoming a top, productive scientist is possible for everyone through effort and practice,” and 2) “Success in science is pretty much related to how much effort a person makes.” Ratings ranged from 1 (strongly disagree) to 5 (strongly agree). In the post-summer survey, students also reported experience of IP during the program and any coping strategies used. In structured progress reports, students wrote about the value of research, cancer prevention, personal growth, and career development from their experiences.

### Analyses

We used a convergent mixed-methods design [35] to analyze and compare qualitative and quantitative data simultaneously. *Qualitative phase.* To gain a nuanced understanding of IP experiences, authors experienced in qualitative analysis (DC, HYL) independently reviewed trainee responses to open-ended questions in the surveys and text from progress reports [36]. Guided by the goal to evaluate workshop effectiveness, we identified key ideas related to IP by conducting a thorough content analysis of the text. After initially creating codes independently, we resolved coding disagreements and then iteratively constructed themes based on codes using constant comparison [37, 38] and analytic induction [39, 40]. *Quantitative phase.* For quantitative analysis, we used a series of repeated-measures ANOVA to examine changes in IP and ability mindset by gender, race/ethnicity, and academic rank. Alpha was set at 0.05. To allow comparison of effect size across the different measures, partial eta^2^, where the effects of other independent variables and interactions are partialled out, was calculated for each of the dependent variables and are reported for significant effects [41]. All methods were carried out in accordance with relevant guidelines and regulations.

## Results

### Qualitative findings

IP manifested as (1) fear about asking for help; (2) comparison of self with others; (3) fear of lack of skills or experience; (4) self-doubt; and (5) uneasiness adjusting to new environments or new tasks, or both (Table 2). Some students recognized having IP experiences (e.g., “*Since the workshop, I am better able to identify and acknowledge when I am feeling like an impostor.”*). Several reported working hard to overcome a potential root cause of impostor feelings: their fear of not knowing enough about their research. Notably, students reported experiencing IP when preparing and delivering elevator speeches and poster presentations, which were new activities for many. Reported by students to be “important,” these were “public performance experiences” for which they expected evaluation by mentors, colleagues, and others.

**Table 2:** Manifestations of IP

Theme	Quotations
Fear about asking for help	“I definitely struggled getting accustomed to my lab and feeling comfortable asking questions and asking for help, which was definitely impostor syndrome because all of my lab members were extremely kind.”
Comparison of self with others	“I have a different career path than many of my peers in CPRTP. I am not as knowledgeable on many topics that my peers are well-rehearsed in due to my focused career path. This made me feel less valuable when discussing academic or social topics with my peers.” “… especially when talking about my project with my mentor since I feel like i actually don’t know anything compared to her. I’ve managed it by reading and informing myself more on the topic.”
Fear of lack of skills or experience	“In laboratory, I had to learn how to do experiments for the first time, and throughout the summer, made many mistakes. This made me feel as though I was inherently incompetent.” “Sometimes I felt as if I could not perform the statistics necessary which made me feel like an impostor.”
Having self-doubt	“I am always in these high-powered meetings where I sit and listen to presentations about results given by professors or postdocs and the discussion these create can go above my head and create feelings of ‘oh I’m not good enough for this kind of thing’ but then I realize, ‘wait a minute, I’m a grad student.”
Uneasiness adjusting to new environments or new tasks, or both	“Being at the largest, and best, cancer center in the country makes me feel that I am on a team bigger than myself, but it’s hard to feel like I belong here—which is probably coming from impostor phenomenon.” “… coming in, I was already wondering why I was picked over all the other qualified people who applied, not completely having a perspective to what people saw in me. The impostor syndrome presentation blew me away and brought a lot of thought to the forefront of my mind.” “… I have often felt this feeling of inadequacy in situations that I earned a position in, but never knew what this feeling was. Learning about the impostor phenomenon gave me the answer I was longing for. Also, learning that this phenomenon is so common gave me comfort that I am not alone, and gave me tools on how to combat and recognize these feelings before they come. Accordingly, I am getting more comfortable talking to the well-established and world-renowned doctors and faculty around the medical center. I now ask them questions that I may have never had the courage to before this program. This opportunity has also reassured me that I am going down the right path.”

Many reported using workshop methods to deal with IP. For example, students reported implementing specific strategies to change their mindset: positive self-talk (e.g., “*remind myself that I am [mostly] not here because of pure luck.*”); refocus of thinking on facts about themselves and the situation (e.g., *“you’re here to learn [otherwise you would not be there in the first place]”*); and recognizing and refuting impostor feelings (e.g., “*I reminded myself that despite those feelings of doubt, there is little to negate the fact that my name is on that poster.*”). They also sought support from mentors, colleagues, and family, recognizing their ability to combat impostor feelings when knowledge gained from the workshop was reinforced during moments of struggle (e.g., “*as I was preparing for my Elevator Speech, I felt a little nervous and my lab member reminded me that I am the expert of this project and I needed to own it.”*).

Multiple benefits from the workshop emerged. In final progress reports, students reported that learning to deal with IP using specific strategies had impact on their career goals (e.g., “*I frequently doubted intelligence and ability because I have not accomplished one of my life goals of becoming a physician. ... My self-confidence has been on a high and I feel like I can accomplish anything if I work hard and enjoy what I do.”).* Students reported appreciating the opportunity to meet other students, working together on IP solutions during the workshop, and learning that having impostor feelings was common. They also valued scheduling the workshop during orientation to help them begin their summers successfully, viewing it as endorsement of its importance and the long-term usefulness of the content shared.

### Quantitative findings

#### IP, mindset changes over time

Scores for IP items from pre-summer surveys indicated in general that student IP feelings were slight to moderate (Table 3). By gender, changes by end of summer in the IP1 and IP2 items were not statistically significant. However, for item IP3 “Sometimes I’m afraid others will discover how much knowledge I really lack,” an interaction between time and gender was statistically significant (*p* = 0.01, partial eta^2^ = .14); specifically, male students reported lower IP feelings and female students reported higher IP feelings after the program. For growth mindset measures, scores for “Success in science is pretty much related to how much effort a person makes” (GM2) improved significantly by the end of summer for all students, regardless of gender, race, or ethnicity (*p*_g_ = .03, partial eta^2^ = .11; *p*_r/e_ = .049, partial eta^2^ = .09), but there were no significant changes by student rank. No changes over the summer reached statistical significance for GM1 by gender, race, ethnicity, or student rank.

**Table 3:** Pre- and Post-summer Mean (SD) Survey Responses for IP and GM Questions by Student Demographics

Variable	IP1			IP2			IP3			GM1			GM2		
	Pre-	Post-	*p*-value	Pre-	Post-	*p*-value	Pre-	Post-	*p*-value	Pre-	Post-	*p*-value	Pre-	Post-	*p*-value
Female	2.33 (1.29)	2.64 (1.25)	p_time=_ 0.81 p_int=_ 0.17	2.00 (0.92)	2.22 (1.04)	p_time=_ 0.57 p_int=_ 0.08	2.30 (1.02)	2.55 (1.06)	p_time=_ 0.23 **p**_**int=**_ **0.01**	3.88 (0.96)	3.82 (0.68)	p_time=_ 0.66 p_int=_ 0.43	3.69 (0.90)	3.81 (0.78)	**p**_**time=**_ **0.03** p_int=_ .13
Male	2.50 (1.40)	2.29 (0.99)		2.07 (1.21)	1.64 (0.75)		2.43 (1.34)	1.79 (0.58)		4.00 (0.96)	4.21 (0.98)		3.36 (1.28)	4.00 (0.56)	
Hispanic	3.14 (1.46)	3.00 (1.29)	p_time=_ 0.53 p_int=_ 0.38	2.43 (1.13)	1.86 (1.07)	p_time=_ 0.81 p_int=_ 0.55	2.86 (1.07)	2.57 (0.79)	p_time=_ 0.81 p_int=_ 0.26	4.14 (0.69)	4.14 (0.38)	p_time=_ 0.96 p_int=_ 0.69	3.43 (1.40)	4.14 (0.69)	**p**_**time=**_ **0.049** p_int=_ 0.70
Black	1.83 (1.27)	2.25 (1.29)		1.55 (0.93)	1.73 (0.65)		1.58 (0.52)	2.08 (0.90)		3.58 (1.17)	3.58 (1.00)		3.36 (1.03)	3.64 (0.81)	
White	2.31 (1.03)	2.08 (1.04)		2.15 (1.07)	2.23 (1.17)		2.54 (1.20)	2.23 (1.01)		4.00 (0.91)	3.77 (0.73)		3.77 (0.73)	3.85 (0.80)	
Asian	2.53 (1.41)	2.93 (1.03)		2.07 (0.88)	2.20 (1.01)		2.53 (1.19)	2.47 (1.19)		4.00 (0.93)	4.27 (0.70)		3.80 (1.01)	3.93 (0.59)	
College	2.50 (1.34)	2.55 (0.96)	p_time=_ 0.25 p_int=_ 0.71	2.05 (1.00)	2.09 (0.87)	p_time=_ 0.80 p_int=_ 0.92	2.55 (1.22)	2.36 (0.90)	p_time=_ 0.72 p_int=_ 0.56	4.09 (0.81)	4.18 (0.73)	p_time=_ 0.89 p_int=_ 0.87	3.48 (1.12)	3.90 (0.70)	p_time=_ 0.59 p_int=_ 0.16
Graduate	2.16 (1.12)	2.32 (1.20)		2.00 (1.09)	1.94 (1.00)		2.00 (1.00)	2.05 (0.97)		3.68 (1.06)	3.68 (0.82)		3.47 (0.91)	3.84 (0.77)	
Medical	2.67 (1.86)	3.17 (1.72)		2.00 (0.89)	2.17 (1.47)		2.67 (0.82)	3.00 (1.27)		4.00 (1.10)	3.83 (0.75)		4.33 (0.82)	3.83 (0.75)	

Significant effects (*p* < 0.05) are bolded. p_time_ = *p*-value of time effect, p_in t_ = *p*-value of interaction effect between time and a predictor. IP1 = “People tend to believe I am more competent than I really am,” IP2 = “I tend to feel like a phony,” IP3 = “Sometimes I’m afraid others will discover how much knowledge I really lack.”GM1 = “Becoming a top, productive scientist is possible for everyone through effort and practice,” GM2 = “Success in science is pretty much related to how much effort a person makes”

#### IP, mindset relationships

The expected significant correlations between the three IP items were found in both the pre- and post-summer surveys, as were correlations between the two growth mindset items, as shown in Table 4. In pre-summer surveys, two IP items (IP2 and IP3) were inversely related to the GM2 mindset measure (r_IP2_ = − 0.40, *p* < .01; r_IP3_ = − 0.32, *p* < .05). By the end of summer, the same correlations had weakened, rendering that between IP3 and GM2 non-significant (r_IP2_ = − 0.35, *p* < .05; r_IP3_ = − 0.12, *p* > .05); this occurred because GM2 scores had increased significantly without change in IP3 scores. This indicated that students’ increased endorsement in the mindset that scientific success relates to efforts made, but without change in their experience of fear of being discovered to have limited knowledge.

**Table 4:** Correlations Between IP and Growth Mindset for Pre- and Post-Summer Survey Responses

		Pre-summer survey response					Post-summer survey response				
		IP1	IP2	IP3	GM1	GM2	IP1	IP2	IP3	GM1	GM2
Pre	IP1	1									
	IP2	0.48^**^	1								
	IP3	0.43^**^	0.74^**^	1							
	GM1	−0.03	−0.22	−0.01	1						
	GM2	0.07	−0.40^**^	−0.32^*^	0.39^**^	1					
Post	IP1	.57^**^	0.15	0.23	−0.02	0.17	1				
	IP2	0.09	0.31^*^	0.53^**^	**−** 0.16	**−** 0.13	0.34^*^	1			
	IP3	0.24	0.26	0.47^**^	**−**0.11	0.08	0.55^**^	0.70^**^	1		
	GM1	0.28	0.06	0.08	0.25	**−**0.11	0.20	0.00	−0.11	1	
	GM2	0.10	**−**0.12	**−**0.11	0.05	0.29	0.21	−0.35^*^	**−0.12**	0.29^*^	1

Significant correlations are bolded, *p < 0.05, **p < 0.01. IP1 = “People tend to believe I am more competent than I really am”, IP2 = “I tend to feel like a phony,” IP3 = “Sometimes I’m afraid others will discover how much knowledge I really lack”; GM1 = “Becoming a top, productive scientist is possible for everyone through effort and practice”; GM2 = “Success in science is pretty much related to how much effort a person makes”

### Disparities between qualitative and quantitative IP measures

We observed differences between quantitative measures of IP and qualitative comments about experiencing IP. In the post-summer survey, 19 participants (16 females) reported higher IP scores but described the usefulness of the workshop and their successful use of learned IP strategies, while 15 other students (9 females) reported lower IP scores and successful use of coping strategies. Two male students reported not having had previous IP experiences in open-ended responses, but reported relatively high pre-summer IP item scores (means = 3.6 ~ 4.7) and one female with relatively low IP scores before and after the program (mean for both = 1.7) reported deriving great benefit from the workshop: “*Being accepted here at all gave me a huge amount of impostor feelings.... However, working through this program has given me a lot of confidence that has really put away a lot of those feelings*.”

## Discussion

Although evidence on interventions that specifically address IP is lacking in the peer-reviewed literature, the development of structured training to help individuals avoid and manage IP threats successfully has been encouraged to prevent the long-term impact of impostor feelings [22]. In academic and professional training settings, such interventions could have a critical impact on trainee resilience and long-term commitment to pursuing research careers by helping them recognize barriers associated with IP and apply strategies to manage IP-related feelings effectively. Importantly, national efforts to foster a diverse STEM or health science workforce cannot be achieved if experiences of IP increase risk of departing from research career paths, especially for those from historically underrepresented groups. Our one-time, brief workshop delivered at the beginning of a summer research program for students helped them learn about IP and the commonality of its experience, and effectively influenced students’ growth mindset and appraisals of their IP coping ability.

A key finding was students’ successful use of strategies learned during the workshop. Quantitative analysis revealed stability in the low to moderate IP scores over the summer, with significant changes in only one measure of IP (IP3, lack of knowledge) by gender (females > males). However, students’ qualitative responses indicated increased ability to cope with IP through learned strategies, and the mechanism appeared to work through students’ activation of growth mindset. Specifically, students concerned about being exposed for their lack of knowledge appeared to have reduced such concern by the end of summer by endorsing a growth mindset about their ability. Importantly, we saw significant increases over the summer in growth mindset (GM2, success in science) for all students. Finally, we observed that qualitative comments did not always align with quantitative measures of IP indicating that sole reliance upon current IP instruments to identify those experiencing IP may not identify all such individuals, especially among diverse groups.

Our results suggest that interventions to address IP may be informed by the vast literature describing successful interventions for managing stress and anxiety [42, 43]. For example, cognitive reappraisal of stressors, which focuses on changing a person’s interpretation of the stressor and therefore changing the stress response (e.g., test anxiety as a performance enhancer vs. inhibitor [44]), has been of growing interest for intervention development and incorporated fixed/growth mindset orientations [45–47], also linked to risk of impostor fear [48]. Importantly, changes in mindset are associated with increases in coping with stressful events but not removing their threat [49]. Similarly, in our workshop evaluation, in spite of positive changes in mindset, impostor feelings remained stable or even increased, but improvement in coping with these feelings was documented.

A major strength of this evaluation was its mixed-methods design that used simultaneously collected qualitative perceptions of student IP experiences for comparison with quantitative metrics developed to measure IP [35]. Other strengths included embedding the workshop within a robust full-time summer research program and workshop effectiveness delivered once, for short duration, and for students diverse by race and ethnicity, gender, and rank. Also, the workshop used a variety of activities to complement didactic presentation of concepts and provide opportunity for students to practice generating strategies and consider how and when to apply them. Working together on these tasks was reported by students to help them engage with the concepts and with one another, thus enhancing their learning experience (Table 1). The project also had limitations. For example, even though the three items used to measure IP were taken directly from two well-known IP scales (i.e., the Harvey, the Leary), we were not allowed to administer the complete scales to establish convergent validity. In addition, since we considered this effort exploratory, we did not correct for multiple tests. Lack of anonymity may have suppressed IP scores in pre-summer surveys because students wanted to control self-presentation before beginning a prestigious summer program, rather than disclose vulnerability to IP. This speculation also supports the disparity we saw between qualitative and quantitative IP measures. Moreover, IP measures in the survey about the workshop at the end of the summer were likely influenced by the overall experience of completing the program. Also, because program leaders served as workshop instructors, student responses may reflect social desirability bias. Finally, the sample size was limited, prohibiting generalization of findings to subgroups.

Many recommendations result from this evaluation. Inconsistent alignment of qualitative comments with quantitative measures of IP calls for further development of tools to measure IP, including for individuals from different backgrounds whose experience may not be accurately characterized by available instruments. Focus should also center on measurement of the ability to manage impostor feelings, as changing the experience of IP may not be feasible to achieve from a short workshop over a brief time period. Exploring relationships between IP, mindset, and coping ability will yield important insight into the mechanisms of coping strategies and inform interventions for IP management and improve career persistence. The role of learning environments in activating IP warrants assessment. Previous research has depicted IP as a personality trait [50–52], but recent findings suggest that aspects of learning environments may increase IP vulnerability in addition to individual attributes [53]. For example, studies have shown connections between IP and workplace harassment, including sexism, sexual abuse, and micro-aggressions among female students and trainees in STEM [54, 55].

## Conclusions

We designed and evaluated a brief IP workshop for students in a summer research program and found evidence that ideas and IP strategies from the workshop were used successfully by students. Many who experience IP are well-accomplished and successful, yet may have high risk for departure from research career paths due to insufficient skills for coping with IP. The implementation of this intervention in research training programs to minimize the impact of IP has promise for recruiting and retaining more research trainees and supporting broader diversity in the STEM and biomedical science workforce.

## Data Availability

Data from coded interviews can be made available upon written request to and approval by the corresponding author and co-authors.
